# Effect of commercial wearables and digital behaviour change resources on the physical activity of adolescents attending schools in socio-economically disadvantaged areas: the RAW-PA cluster-randomised controlled trial

**DOI:** 10.1186/s12966-021-01110-1

**Published:** 2021-04-12

**Authors:** Nicola D. Ridgers, Anna Timperio, Kylie Ball, Samuel K. Lai, Helen Brown, Susie Macfarlane, Jo Salmon

**Affiliations:** 1 Institute for Physical Activity and Nutrition (IPAN), School of Exercise and Nutrition Sciences, Deakin University, Geelong, Australia; 2 Learning Futures, Deakin University, Burwood, Victoria Australia

**Keywords:** Intervention, Adolescents, Physical activity, Wearable activity tracker, Social media

## Abstract

**Background:**

There has been increasing interest in using wearable activity trackers to promote physical activity in youth. This study examined the short- and longer-term effects of a wearable activity tracker combined with digital behaviour change resources on the physical activity of adolescents attending schools in socio-economically disadvantaged areas.

**Methods:**

The Raising Awareness of Physical Activity (RAW-PA) Study was a 12-week, multicomponent intervention that combined a Fitbit Flex (and accompanying app), and online digital behaviour change resources and weekly challenges delivered via Facebook. RAW-PA was evaluated using a cluster-randomised controlled trial with 275 adolescents (50.2% female; 13.7 ± 0.4 years) from 18 Melbourne secondary schools (intervention *n* = 9; wait-list control group *n* = 9). The primary outcome was moderate- to vigorous-intensity physical activity (MVPA), measured using hip-worn ActiGraph accelerometers. The secondary outcome was self-reported physical activity. Data were collected at baseline, 12-weeks (immediately post-intervention), and 6-months post-intervention (follow-up). Multilevel models were used to determine the effects of the intervention on daily MVPA over time, adjusting for covariates.

**Results:**

No significant differences were observed between intervention and wait-list control adolescents’ device-assessed MVPA immediately post-intervention. At 6-months post-intervention, adolescents in the intervention group engaged in 5 min (95% CI: − 9.1 to − 1.0) less MVPA per day than those in the wait-list control group. Males in the intervention group engaged in 11 min (95% CI: − 17.6 to − 4.5) less MVPA than males in the wait-list control group at 6-months post-intervention. No significant differences were observed for females at either time point. For self-reported physical activity, no significant effects were found at 12-weeks and 6-months post-intervention.

**Conclusions:**

Combining a wearable activity tracker with digital behaviour change resources and weekly challenges did not increase inactive adolescents’ accelerometer-derived and self-reported physical activity levels immediately post-intervention. This contrasts previous research that has suggested wearable activity tracker may increase youth physical activity levels in the short-term. Lower engagement in MVPA 6-months post-intervention was observed for males but not for females, though it is unclear why this finding was observed. The results suggest wearable activity trackers, in combination with supporting materials, may not be effective for increasing physical activity levels in adolescents.

**Trial registration:**

ACTRN12616000899448. Australian and New Zealand Clinical Trials Registry. Registered 7 July 2016.

**Supplementary Information:**

The online version contains supplementary material available at 10.1186/s12966-021-01110-1.

## Background

It has been well documented that higher levels of physical activity benefit adolescents’ physical, social, and mental health [1, 2]. Despite this, 80% of adolescents aged 13–15 years old engage in less than the recommended 60 min of moderate- to vigorous-intensity physical activity (MVPA) every day [3]. Steep declines in physical activity have been shown to occur during adolescence [4], with greater decreases observed among those living in socioeconomically disadvantaged areas [5]. As such, there is a need to develop and test strategies that aim to increase physical activity levels in adolescents living in socioeconomically disadvantaged areas, who are an under-represented group in physical activity interventions [6].

Wearable activity trackers have been the leading fitness trend in recent years [7], and widespread uptake has been reported within different population age groups [7, 8]. Unsurprisingly, there has been considerable interest among researchers and practitioners in the potential that wearable trackers available to consumers offer for physical activity promotion [9, 10]. Rapid advances in technology have resulted in the automation of real-time activity tracking against individualised goals and public health recommendations, enabling the wearer to self-monitor their activity over a prolonged period of time [11, 12]. Self-monitoring has been well established as an effective behaviour change technique in promoting adoption of targeted health behaviours such as physical activity [13]. When wearable physical activity trackers are combined with an accompanying app and/or website platform, this ‘self-monitoring system’ provides an individual with access to a range of features and functions that have been shown to align with up to 26 different behaviour change techniques that are known to be effective [13–15].

Wearable activity trackers have generally been shown to have acceptable validity for measuring steps [16], but little research has examined whether these devices are effective in increasing physical activity levels [11, 17, 18], or how they are used within interventions [9]. In adults the research findings are mixed, with some studies suggesting wearable activity trackers may increase activity levels, yet others suggesting activity declines after initial short-term increases [19–21]. Of the few studies conducted with adolescents, wearable activity trackers have been combined with additional strategies such as goal setting [22, 23], incentives [24], or online educational programs [25] to encourage and support engagement in physical activity. However, the majority of studies have been feasibility pilot interventions delivered over a short time period, lacking longer-term follow-up assessments, involving small samples, and few included adolescents living in socioeconomically disadvantaged areas [11, 18]. Combining social media platforms and online programs alongside wearable trackers is a promising approach for promoting physical activity in adolescents, since these strategies can reach a large proportion of the target audience due to high home internet availability, may motivate participants, and can provide information to educate and upskill individuals in behaviour change techniques [26–28]. However, to date few significant effects on physical activity have been reported [29, 30].

The aim of this study was to examine the 12-week intervention and 6-month maintenance effects of a wearable activity tracker combined with digital behaviour change resources on the physical activity of adolescents attending schools in socio-economically disadvantaged areas.

## Methods

### Study design and participants

The Raising Awareness of Physical Activity (RAW-PA) Study was a 12-week multi-component intervention that combined a wearable activity tracker and supporting digital materials that targeted adolescents attending schools in socioeconomically disadvantaged areas. The RAW-PA Study rationale and protocols have been reported in detail elsewhere [31]. The CONSORT statement for cluster randomised controlled trials [32] and the TIDieR checklist [33] are used in the reporting of this study. The CONSORT checklist and the TIDieR checklist are provided in Additional file 1 and 2, respectively. In brief, the intervention was evaluated using a cluster-randomised controlled trial with measures conducted at baseline, immediately post-intervention (12 weeks), and 6-months post-intervention. Schools within ~ 60 km of Deakin University’s Burwood Campus with a Socio-Economic Indexes for Areas (SEIFA) [34] score of ≤5 (lowest 50%) were eligible to participate. Eighteen secondary schools (42% response rate) provided written informed Principal consent to participate in the study. Following baseline assessments, schools were paired based on SEIFA score and size and randomly allocated to either the intervention (9 schools) or wait-list control (9 schools) group using a computer-based random number generator [31]. An independent researcher who was not involved in the study conducted the randomisation process.

Students were eligible to participate in the intervention if they were in Year 8, at least 13 years old, had access to the internet outside of school (e.g., via smartphone or home internet), self-reported that they did not engage in regular physical activity/sport, did not meet current physical activity guidelines, and reported that they had not previously owned or used a wearable activity tracker. Students were provided recruitment information by a liaison teacher in each participating school, and eligibility was determined based on a checklist completed by the students and their parents. In total, 280 adolescents (20% of students in Year 8 at participating schools) self-identified as meeting the inclusion criteria and provided written informed parental consent and student assent to participate in the study. Ethics approval for this study was received from the Deakin University Human Research Ethics Committee (2016–179) and the Victorian Department of Education and Training. The study is registered with the Australian and New Zealand Clinical Trials Registry (No: ACTRN12616000899448).

### Power calculation

The sample size was based on detecting differences in daily MVPA between the intervention and wait-list control students immediately post-intervention [31]. Based on an initial sample size of 300 students (150 per group), and assuming an α of 0.05, power of 80%, and a 30% dropout rate, it was calculated that this study would be powered to detect a difference in daily MVPA of 7.9 min between intervention and wait-list control students. A difference of 10 min has previously found to be clinically meaningful [35]; a difference of this magnitude that this study was powered to detect.

### Intervention

RAW-PA was informed by and developed using participatory research principles. Adolescents in Year 8 were involved in designing and reviewing the intervention approach, components, and materials. A detailed description of this process and the intervention has been previously reported [31, 36]. In brief, students provided their thoughts on how to integrate wearable activity trackers into a physical activity intervention. Feedback was provided on the format and content of behaviour change resources, for example, and developed materials were refined further based on additional suggestions [31]. RAW-PA was grounded in Behavioural Choice Theory [37] and Social Cognitive Theory [38], and targeted key intrapersonal (e.g., enjoyment, self-efficacy) and interpersonal (e.g., teachers, peers) influences on health behaviour (see [31] for details). The intervention included the following components: a Fitbit Flex and accompanying app (provided free of charge); interactive weekly individual and/or team ‘missions’ and behaviour change resources accessible via a private, researcher-moderated Facebook group, and alerts (e.g. text messages) to new online content [31]. The weekly missions were posted in the Facebook group at the start of each week by the research team. The missions were designed to help students learn and develop behaviour change techniques (e.g. goal setting, self-monitoring, self-efficacy) that have been established as effective for changing health behaviours [13], and focused on increasing physical activity by incorporating more movement into daily life. There were 12 weekly missions including, for example, “Pair Up!”, which focused on engaging in physical activity with friends, and “Step it Up!”, which focused on evaluating and adjusting set goals [31]. The accompanying behaviour change resources (e.g. infographics, short videos) were matched to the weekly mission to communicate and reinforce key messages. The online mode of delivery of the missions and resources via the Facebook group ensured that the information was readily available and enabled students to engage with the content in their own time. Alerts to new content were sent 2–3 times a week. The private Facebook group was also intended to be a supportive social forum for sharing experiences with other students at intervention schools and the research team during the intervention. For practical reasons, RAW-PA was delivered across three different waves between September 2016 and October 2017, with schools participating in one wave. Participants in the paired schools (wait-list control) received the intervention at the completion of the 6-month post-intervention assessments.

### Wearable activity tracker

The intervention used the Fitbit Flex (~AUS $100), which is a wrist-worn monitor that provides estimates of steps taken, physical activity intensities, estimated energy expenditure, and sleep duration. Feedback on steps taken is provided to the wearer via a visual display consisting of five light emitting diode (LED) indicator lights which light up as the individual progresses towards their individualised daily goal created during the Fitbit Flex set-up process (one light represents 20% of the goal achieved). To view more detailed information on collected data, participants were required to sync the device with their online Fitbit account. This free account is accessed using the Fitbit smartphone app or via the Fitbit website. The Fitbit Flex requires charging approximately every 5 days and stores data for up to 7 days without being synced to the user’s account [36]. At the start of the intervention, research staff assisted students in setting up the device and their Fitbit profile, and provided initial information about how to charge and sync the device. This information was also provided within the packaging of the device. At the end of the 12-week intervention period, adolescents in the intervention group and wait-list control kept the Fitbit Flex as compensation for participation.

### Measures

A survey containing questions about demographic characteristics (e.g., marital status, employment status, education) was completed by the adolescents’ parents at baseline. The highest reported parental education was used as a proxy-measure of socio-economic status and was classed as low (some high school attendance or less), medium (high school or trade certificate completed), or high (tertiary education).

Trained research assistants completed data collection with adolescents in schools at each time point using standardised protocols unless otherwise specified. Research staff were not blinded to treatment allocation at 12-week post-intervention or at 6-month post-intervention.

#### Accelerometry

Students’ physical activity levels were objectively assessed for 8 consecutive days using a hip-mounted ActiGraph GT3X+ accelerometer (Pensacola, FL, USA) at each time point. The accelerometer has acceptable reliability and validity for use in this population [39]. Students were instructed to wear the accelerometer during waking hours and only remove it for water-based activities and sleeping. Raw data were sampled at 30 Hz and downloaded into 15 epochs for analysis. Age-specific thresholds were used to determine time spent in moderate- to vigorous-intensity physical activity (MVPA [40];). Non-wear time was defined as 60 min of consecutive zeroes [41]. A valid day was defined as ≥8 h on weekdays and ≥ 7 h on weekends to maximise sample retention [42]. Data at each time point were included for analyses if the accelerometer had been worn for at least 3 days [42]. Complete (valid) accelerometer data were collected from 246 students at baseline. Immediately post-intervention and 6-month post-intervention, valid accelerometry data were collected from 198 and 193 students, respectively.

#### Survey data

Students completed a questionnaire on an iPad at each time point that included questions concerning demographic information (e.g. sex, age, date of birth). Surveys were administered at the student’s school by trained research assistants. If a student was absent on the scheduled data collection visit, they completed a hard copy survey on their return to school under the supervision of their teacher. The surveys included assessments of leisure-time physical activity and sedentary time, potential mediators of behaviour change, and process measures (e.g. acceptability, appeal of RAW-PA intervention [31]). A comprehensive process evaluation has been undertaken and is published elsewhere [43]. For the purpose of this study, questions that asked students to self-report the number of days (0–7) on which they had engaged in MVPA for a total of 60 min per day over the past 7 days and over a typical week were analysed [44]. A definition of MVPA and examples of physical activities were provided, and students were asked to sum the time spent in MVPA each day. The two items responses were averaged for use in the analyses [44]. These items have been validated for use with adolescents [44, 45].

### Statistical analyses

Descriptive analyses were initially conducted for all measured variables. Analyses were performed using the intention-to-treat principle, with students analysed according to the group to which their school was randomised [46]. To determine the effects of the intervention on daily MVPA and whether effects were maintained over time, multilevel models were used. As multilevel model analyses are robust to missing data points, do not require complete data sets, and can estimate effects over time using incomplete data sets, all collected data that met the inclusion criteria at each time point were used in the analyses [47, 48]. Missing data were assumed to be missing at random. A three-level model was used: time point (Level 1); adolescent (Level 2); and school (Level 3). The random structure considered random intercepts. Time (12 week post-intervention, 6-month post-intervention) and intervention group (intervention, wait-list control) were included as fixed factors. The initial model was adjusted for baseline values and accelerometer wear time (when accelerometer data were analysed). The final model additionally adjusted for sex, baseline age, and wave of data collection. A sex by time interaction term was calculated and where significant, sub-group analyses by sex were undertaken. A sensitivity analysis was conducted for completers (i.e. participants who provided complete data at baseline, immediately post-intervention, and 6-month post-intervention). Statistical significance was set at *p* < 0.05. All analyses were conducted using Stata SE v15 (StataCorp, Texas, USA).

## Results

**Fig. 1:**
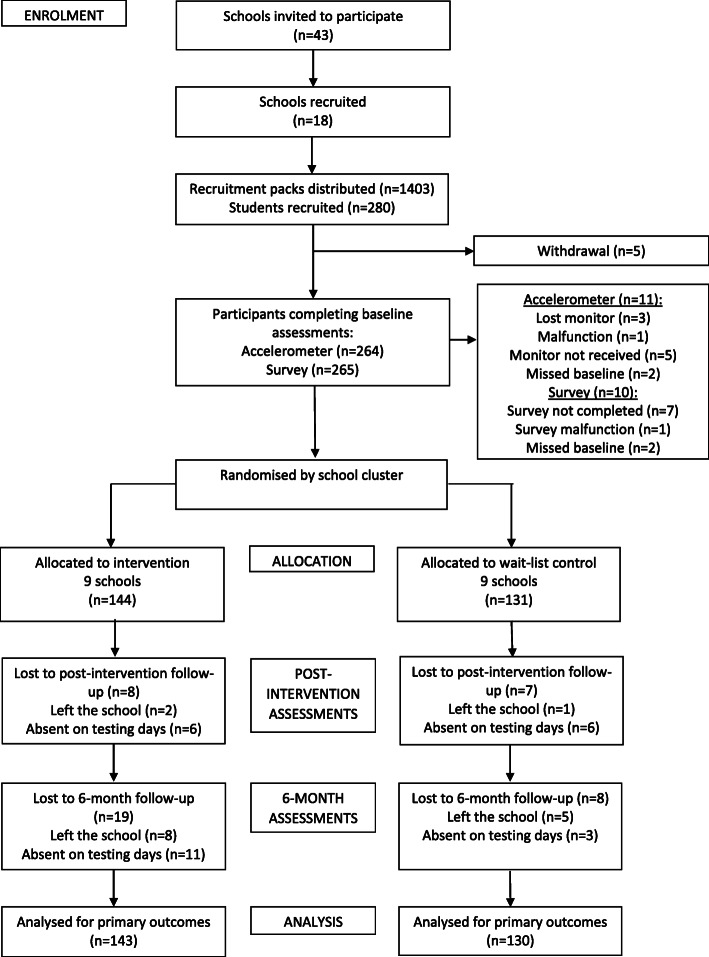
Flow of adolescents throughout the study

The flow of the participants through the study is shown in Fig. 1. Five students withdrew from the study prior to baseline assessments, resulting in a sample of 275 adolescents (51.6% female). This represented 91.7% of the target sample. At baseline, 264 students (52.3% female) received an accelerometer and 265 students (52.1% female) completed a survey (Fig. 1). In total, 267 parents returned a survey. Based on the 258 parents who provided information about parental education, 32.2 and 37.6% were classed as low and medium socio-economic status, respectively. Descriptive baseline information for the adolescents is presented in Table 1.

**Table 1:** Baseline characteristics of study sample (mean ± SD; unless specified)

	Intervention	Wait-list Control	Whole sample
Age (years)	13.8 (0.4)	13.7 (0.4)	13.7 (0.4)
Female students (%)	48.6	56.4	52.3
Moderate- to vigorous-intensity physical activity (min/d)	36.6 (19.3)	39.1 (18.4)	37.8 (18.9)
Self-reported physical activity (days)	2.8 (1.8)	2.8 (1.7)	2.8 (1.8)

### Main analyses

Table 2 reports the intervention effects (partially-adjusted and fully-adjusted models) on MVPA and self-reported physical activity for the intervention group compared to the wait-list control group immediately post-intervention and at 6-month post-intervention. Significant sex by time interactions were found for MVPA and self-reported physical activity (*p* < 0.05). No significant intervention effects for daily MVPA were found immediately post-intervention in the whole sample and for males and females. At 6-month post-intervention, students in the intervention group engaged in significantly less MVPA than students in the wait-list control group (adjusted model: −5.0 min; 95% CI: −9.1 to −1.0). Sub-group analyses found that males in the intervention group engaged in significantly less MVPA than males in the wait-list control group (adjusted model: −11.0 min; 95% CI: −17.6 to −4.5) at 6-month post-intervention. No significant intervention effects were observed for females’ MVPA at 6-month post-intervention. For self-reported physical activity, no significant intervention effects were found immediately post-intervention or at 6-month post-intervention for the whole sample or in the sub-group analyses.

**Table 2:** Intervention effects on physical activity outcomes by intervention group immediately post-intervention and 6-month post-intervention

	Partially-adjusted model^b^			Fully-adjusted model^c^		
	β	95% CI	***p***-value	β	95% CI	***p***-value
*Accelerometry*						
Immediately post-intervention MVPA (min/d)^a^						
Whole sample	0.7	−3.47 to 4.89	0.76	0.5	−3.54 to 4.50	0.81
Males	−0.9	−7.14 to 5.37	0.78	−0.1	−6.45 to 6.16	0.96
Females	−0.02	−5.16 to 5.11	0.99	0.4	−4.66 to 5.56	0.86
6-month post-intervention MVPA (min/d)^a^						
Whole sample	**−4.4**	**−8.60 to -0.24**	**0.04**	**−5.0**	**−9.07 to −0.96**	**0.02**
Males	**−10.9**	**−17.46 to −4.38**	**0.001**	**−11.0**	**−17.57 to −4.48**	**0.001**
Females	−0.3	−5.38 to 4.78	0.91	−0.03	−5.06 to 5.01	0.99
*Self-report*						
Immediately post-intervention PA (days)^a^						
Whole sample	0.2	−0.17 to 0.62	0.26	0.2	−0.17 to 0.62	0.27
Males	0.1	−0.54 to 0.71	0.79	0.09	−0.54 to 0.71	0.79
Females	0.3	−0.16 to 0.82	0.19	0.4	−0.12 to 0.85	0.14
6-month post-intervention PA (days)^a^						
Whole sample	0.2	−0.25 to 0.55	0.46	0.1	−0.26 to 0.54	0.49
Males	0.02	−0.61 to 0.65	0.95	0.02	−0.61 to 0.66	0.95
Females	0.2	−0.26 to 0.73	0.35	0.3	−0.21 to 0.77	0.27

^a^Difference between intervention and control group; significant differences are bolded

*CI* Confidence interval, *MVPA* Moderate- to vigorous-intensity physical activity, *PA* Physical activity

^b^Partially adjusted model: adjusted for baseline outcome values and accelerometer wear time (for MVPA outcome only)

^c^Fully-adjusted model: additionally adjusts for baseline age, sex, wave of data collection

### Sensitivity analyses

The pattern of results for MVPA (*n* = 150) and self-reported physical activity (*n* = 221) were similar when comparing results for completers only with the respective results in the main analyses (Additional file 3).

## Discussion

This study aimed to examine the 12-week post-intervention and 6-month maintenance effects of a wearable activity tracker combined with behaviour change resources on inactive adolescents’ physical activity. No significant intervention effects were found for accelerometer-assessed daily MVPA immediately post-intervention (12-weeks). At 6-months post-intervention, adolescents in the intervention group engaged in significantly less MVPA than those in the wait-list control group. No significant intervention effects were found for self-reported physical activity immediately post-intervention or at 6 months post-intervention.

Little research has examined the effects of wearable activity trackers on youth physical activity, either alone or in combination with other strategies. Despite multicomponent strategies showing promise for increasing physical activity in different populations [18], no intervention effects were found immediately post-intervention in this study. This is largely consistent with previous research in children and adolescents that has used wearable activity trackers either alone or in combination with different strategies (e.g. social media, face-to-face education sessions) in clinical and non-clinical populations [22, 24, 29, 30, 49]. It should be noted though that only one study included adolescents living in socioeconomically disadvantaged areas [24]. Moreover, the majority typically examined the feasibility, acceptability and preliminary efficacy of different wearable activity tracker interventions in children and adolescents, which may not have been sufficiently powered to detect changes in daily MVPA [11, 18].

In contrast to a number of previous studies [23, 24, 49, 50], RAW-PA was designed to be delivered online, and adolescents could access the behaviour change resources in their own time in a flexible, interactive way [31]. The behaviour change resources and materials were co-designed with the target population (adolescents), low-cost strategies were incorporated into the intervention to facilitate implementation, and the online delivery was utilised given its reach, accessibility, and the potential for wider scale up [31, 43]. However, the lack of significant findings immediately post-intervention suggests that the behaviour change resources and strategies provided online may not have been sufficient for increasing adolescents’ activity levels. Indeed, these results indicate that digitally delivered interventions may need to be supplemented with more intensive or face-to-face support to increase activity levels, particularly as engagement with the Facebook group and the weekly challenges/missions were low during the intervention [43]. Such strategies may include the provision of tailored physical activity advice (e.g. how to be active), the development of support structures, or for content to be delivered in partnership with teachers or parents [18, 24, 50, 51]. Studies that have only provided wearable activity trackers found that such minimalist strategies may initially increase awareness of activity levels but may not be sufficient for increasing activity levels [22, 52]. Moreover, it is possible that the wearable activity tracker itself may have contributed to the lack of intervention effects. Despite pilot testing the Fitbit Flex with adolescents and addressing potential barriers to use within the RAW-PA intervention [31, 36], the Fitbit Flex has a limited display and requires the use of the accompanying app to obtain information concerning activity levels. Previous research has shown that utilising mobile phone data to view such data in an app was a barrier to use, regardless of socioeconomic status [36, 53]. Furthermore, the RAW-PA implementation evaluation found that while the Fitbit Flex was perceived as being easy to use [43], students considered it required effort to use it (e.g. charge and sync [54]). This may have resulted in a lack of engagement by the adolescents with the specific physical activity feedback provided by the Fitbit Flex as intended during the intervention, which in turn could explain the null effects observed for daily MVPA.

The lower MVPA engagement in the intervention group at 6-months post-intervention is attributable to the declines observed in males. This contrasts with findings of a previous study that showed significantly lower sedentary time at 8-months follow-up for males when a wearable activity tracker was combined with web-based tailored physical advice [30], though interestingly this change did not result in increased physical activity of any intensity. Notably, neither the current study or that of Slootmaker and colleagues [30] found any effects on females’ activity levels 6- and 8-months post-intervention, respectively, suggesting that wearable activity trackers may have limited effects on females’ physical activity levels. To date, only one study has reported lower MVPA engagement after using wearable activity trackers, albeit over a shorter time period [55]. Kerner and colleagues found declines in MVPA of 9.5 min per day after adolescents were provided with a Fitbit Charge for 5 weeks, attributing this change to lowered autonomous motivation [55]. It is possible that wearable activity trackers may negatively impact motivation to be active, particularly if the wearer fails to achieve a set activity goal [56] and focuses on the physical activity outcome (e.g. total steps) rather than the process [57, 58]. It is difficult to explain, however, whether this may have resulted in changes at 6-months post-intervention but not immediately post-intervention in the present study, particularly as the implementation evaluation found that few males reported wearing their Fitbit Flex in the last week of the intervention [43]. It may be that the use of the wearable activity tracker resulted in a loss of intrinsic motivation over time, and that the devices instead created a dependence on achieving outcomes and rewards [59], with these effects manifested at 6-months post-intervention. There is a need for studies utilising wearable activity trackers to examine longer-term effects on physical activity levels after the conclusion of an intervention [60]. This would provide important insights into whether use of this technology may have unintended negative effects on adolescents’ physical activity over time, and what factors may explain any observed changes.

No effects were observed for self-reported physical activity either immediately or at 6-month post-intervention. This is consistent with the findings of Bronikowski and colleagues [56] who examined whether specific or ‘do your best’ goals increased youth physical activity levels, assessed using the same self-report measure [44], albeit over an 8-week period. Previous research has demonstrated that inactive adolescents tend to overestimate their activity levels and intensity, and may be unware of how much daily activity that they need to engage in [61]. It has been suggested that the feedback provided by the Fitbit Flex may increase adolescents’ awareness of their activity levels [36, 62], which may subsequently encourage them to reflect about the meaning of these data and whether there were any discrepancies between how active they thought they were and actually were [51]. This reflective process may have resulted in little change in self-reported physical activity levels post-intervention. However, there are other potential explanations for the lack of significant findings. For example, while some studies in youth have shown that wearable activity trackers increase motivation to be active, at least in the short-term [63, 64], others have questioned whether increased awareness results in changes in activity levels [52, 62, 65]. Moreover, despite the behaviour change resources and delivery strategy being developed using participatory approaches [31], the implementation evaluation suggested that the delivered intervention may not have met the needs or expectations of the adolescents as engagement with the Facebook group and materials declined over time [43]. This is consistent with a recent study that showed no effects on adolescents’ physical activity despite considerable input from the target group [66]. There is a need for further research to establish the impact of wearable activity trackers, either alone or in combination with additional strategies, on adolescents’ physical activity levels, and examine what factors may be critical for changing behaviour.

This was one of the first studies to examine the effect of combining wearable activity trackers and behaviour change resources on inactive adolescents living in areas of socio-economic disadvantage. Strengths of the study include the cluster-randomised controlled trial design, the grounding of the intervention in theory, the inclusion of both device-based and subjective assessments of physical activity, and delivering the intervention in an ecologically valid setting [67]. However, several limitations should be acknowledged. Firstly, the Fitbit Flex provided limited feedback to participants, which meant that they needed to engage with the app or website to view their activity data in detail [54]. This may have impacted on their ability to self-monitor activity levels in real-time and ascertain progress against set-goals. Second, while the retention rate was relatively high in this study, compliance with the accelerometer measure was low. Complete valid data were provided by approximately half of the sample, though this is similar to previous trials [66]. Third, recruitment into the study was based on self-reporting against inclusion criteria. It is not known how many adolescents were eligible to participate in the study, and those who agreed to participate may not necessarily represent the wider population.

## Conclusion

Combining a wearable activity tracker with online digital behaviour change resources was not effective in increasing the physical activity of adolescents attending schools in socio-economically disadvantaged areas immediately post-intervention or at 6-month post-intervention compared to wait-list control participants. Lower engagement in MVPA 6-months post-intervention was observed for males but not for females, though it is unclear why this finding was observed. Future research is needed to better understand whether wearable activity trackers combined with appropriately engaging digital resources can effectively promote physical activity for adolescents, particularly for males.

## Supplementary Information

**Additional file 1.** RAW-PA consort statement.

**Additional file 2.** The TIDieR (Template for Intervention Description and Replication) Checklist for RAW-PA.

**Additional file 3: Table S1.** Completer’s analysis of intervention effects on physical activity outcomes by intervention group post-intervention and 6-month follow-up.

## Data Availability

The datasets generated and/or analysed during the current study are not publically available due to ethics board requirements but are available from the corresponding author on reasonable request and pending approval from the relevant ethics committees.
