# Supplementary material for: Effect of commercial wearables and digital behaviour change resources on the physical activity of adolescents attending schools in socio-economically disadvantaged areas: the RAW-PA cluster-randomised controlled trial
Source: Int J Behav Nutr Phys Act. 2021 Apr 12;18:52. doi: 10.1186/s12966-021-01110-1 (PMC8042874; doi:10.1186/s12966-021-01110-1)
Supplement: Supplementary file 3 — Additional file 3: Table S1. Completer’s analysis of intervention effects on physical activity outcomes by intervention group post-intervention and 6-month follow-up. [file 12966_2021_1110_MOESM3_ESM.docx]

**Supplement Table 1: Completer’s analysis of intervention effects on physical activity outcomes by intervention group post-intervention and 6-month follow-up**

|  | **Partially-adjusted model** | | | **Fully-adjusted model** | | |
| --- | --- | --- | --- | --- | --- | --- |
| ­ | **β** | **95% CI** | **p-value** | **β** | **95% CI** | **p-value** |
| *Accelerometry* |  |  |  |  |  |  |
| Post-intervention MVPA (min/d)* |  |  |  |  |  |  |
| Whole sample | -0.78 | -5.85 to 4.35 | 0.77 | -1.2 | -6.16 to 3.75 | 0.63 |
| Males | -2.0 | -10.14 to 6.17 | 0.63 | -1.2 | -9.44 to 7.01 | 0.77 |
| Females | -2.5 | -8.70 to 3.62 | 0.42 | -2.0 | -8.03 to 4.00 | 0.51 |
| 6-month follow-up MVPA (min/d)* |  |  |  |  |  |  |
| Whole sample | -4.6 | -9.70 to 0 50 | 0.08 | **-5.4** | **-10.34 to -0.42** | **0.03** |
| Males | **-10.2** | **-18.35 to -2.03** | **0.01** | **-10.5** | **-18.68 to -2.21** | **0.01** |
| Females | -2.7 | -8.85 to 3.46 | 0.39 | -2.1 | -8.07 to 3.96 | 0.50 |
|  |  |  |  |  |  |  |
| *Self-report* |  |  |  |  |  |  |
| Post-intervention PA (days)* |  |  |  |  |  |  |
| Whole sample | 0.2 | -0.25 to 0.61 | 0.41 | 0.2 | -0.24 to 0.61 | 0.39 |
| Males | -0.01 | -0.68 to 0.67 | 0.99 | -0.01 | -0.67 to 0.67 | 0.99 |
| Females | 0.3 | -0.20 to 0.88 | 0.21 | 0.4 | -0.12 to 0.95 | 0.13 |
| 6-month follow-up PA (day)* |  |  |  |  |  |  |
| Whole sample | 0.1 | -0.28 to 0.57 | 0.50 | 0.1 | -0.28 to 0.57 | 0.52 |
| Males | -0.01 | -0.69 to 0.66 | 0.97 | -0.01 | -0.68 to 0.66 | 0.98 |
| Females | 0.3 | -0.28 to 0.80 | 0.35 | 0.3 | -0.22 to 0.85 | 0.24 |

*Difference between intervention and control group; significant differences are bolded

CI = Confidence interval; MVPA = Moderate- to vigorous-intensity physical activity

Complete accelerometer data collected from 150 students: Intervention group: males = 31, females = 33; wait-list control group: males = 34, females = 52.

Complete survey data collected from 221 students: Intervention group: males = 56, females = 57; wait-list control group: males = 48, females = 60.
